# The role of cesarean section surgical techniques in the prevention of isthmocele formation: retrospective cohort study

**DOI:** 10.1007/s00404-026-08359-6

**Published:** 2026-03-21

**Authors:** Jule Eriç Horasanlı, Fatih Akkuş

**Affiliations:** 1 Department of Obstetrics and Gynecology, Faculty of Medicine, Necmettin Erbakan University, Konya, Turkey; 2 Division of Perinatology, Department of Obstetrics, Gynecology, Kütahya City Hospital, Kütahya, Turkey

**Keywords:** Cesarean section, Sutures, surgical, Postoperative complications, Uterus/surgery, Ultrasonography

## Abstract

**Key message:**

Compared with conventional locked double-layer closure, an unlocked double-layer, endometrium-sparing closure was associated with lower isthmocele prevalence, greater residual myometrial thickness, and fewer postcesarean symptoms. If these findings are confirmed in prospective studies, adopting this closure approach could help to reduce morbidity related to the niche in routine cesarean practice.

**Abstract:**

**Background:**

Isthmocele, a cesarean scar defect associated with abnormal bleeding, pelvic pain, and diminished fertility, is becoming more well known as a result of the increasing prevalence of cesarean sections. Although the optimal closure technique is still a topic of debate, it is regarded as the most modifiable factor in the prevention of isthmocele.

**Objective:**

To compare the effects of the traditional locked double-layer uterine closure technique with the new unlocked double-layer, endometrium-protective technique on isthmocele incidence, residual myometrial thickness (RMT), and post-CS symptoms.

**Methods:**

From March 2023 to January 2025, a total of 180 women (97 conventional and 83 novel) who underwent low-segment cesarean sections at a tertiary care center were included in a retrospective comparative cohort study. At 12–24 weeks postpartum, residual myometrial thickness (RMT) and isthmocele presence were evaluated using saline-infusion sonohysterography. Independent predictors of RMT and isthmocele were identified through binary logistic and multiple linear regression analyses.

**Results:**

The new method was linked to less need for additional hemostatic sutures (2.4% vs. 11.3%; OR = 5.18), as well as a shorter operating time (26 vs. 33 min, *p* = 0.001). Compared with the new lockless double-layer technique, traditional locked double-layer closure was associated with a significantly higher risk of isthmocele (39.2% vs. 7.2%; OR = 8.27, 95%CI 3.28–20.85; *p* = 0.001). Additionally, the mean RMT was greater (13.91 vs. 10.18 mm, *p* = 0.001). While linear regression connected the novel technique and higher parity to greater RMT and preoperative anemia to decreased healing, logistic regression found that suture technique was the only independent predictor of isthmocele. The novel group had significantly lower rates of postmenstrual spotting, dysmenorrhea, and chronic pelvic pain (*p* = 0.001 for all).

**Conclusions:**

Compared to the traditional locking technique, the locking, double-layer, endometrium-sparing uterine closure method, in which the first layer of the uterine incision is continuously sutured without locking and the second layer is reinforced with a continuous ‘U’-shaped suture, results in a statistically significant reduction in isthmocele formation, increased residual myometrial thickness, shorter operative time, and fewer niche-related symptoms. This method appears to improve short-term uterine healing and niche-related symptoms; however, its impact on future reproductive and obstetric outcomes remains unknown and requires prospective validation. However, multicenter studies with extended follow-up periods are warranted to confirm these findings.

**Supplementary Information:**

The online version contains supplementary material available at 10.1007/s00404-026-08359-6.

## Introduction

The cesarean section (CS) is the most frequently performed surgery among women [1]. The rising rates of CS inevitably lead to long-term obstetric and gynecological complications. One possible complication is the development of an isthmocele, which is basically a small indentation at the site of a C-section scar that is at least 2 mm deep [2]. Synonyms commonly used in the literature to describe this defect include uterine scar defect, niche, cesarean scar defect, uterine diverticulum, pouch, and sacculation [3]. The development of an isthmocele is often a consequence of poor healing at the uterine incision site [4]. The prevalence of isthmocele has been reported to range from 24 to 70% when assessed using ultrasonographic examination, and it can increase between 56 and 84% with saline-infusion sonography following one or more cesarean sections (CS) [5]. Although several theories have been proposed regarding the development of isthmocele, there is still no definitive consensus [5–8]. Although there is no universally accepted gold standard for diagnosis, the modified Delphi consensus by Jordans et al. [2] suggested that gel or saline sonography serves as a valuable complement to conventional ultrasound in identifying this condition. They characterized an isthmocele as an anechoic defect within the myometrium of the lower uterine segment measuring at least 2.0 mm in depth, whereas earlier studies had used a threshold of 1 mm [9]. Thurmond et al. hypothesized that isthmocele may cause postmenstrual spotting due to menstrual blood collecting in the uterine scar defect [10]. The most common symptoms associated with isthmocele include menstrual bleeding, dysmenorrhea, dyspareunia, chronic pelvic pain, and infertility [4]. In addition, there is an increased risk of scar pregnancies, placentation abnormalities, and uterine rupture in future pregnancies [11]. Given the rising rates of cesarean deliveries worldwide, the current prevalence of isthmocele and its associated complications should not be overlooked. Although, hysteroscopic and laparoscopic isthmocele repair procedures have demonstrated high success rates [12]. Preventing isthmocele formation during CS should be the primary goal. Recent evidence suggests that the method of uterine closure can significantly impact subsequent scar morphology. A meta-analysis due to be published in 2025 concluded that there are measurable differences in niche formation rates after cesarean delivery between single-layer and double-layer closure [13].

The uterine closure technique is considered the most significant factor influencing isthmocele formation, but other contributing factors include the proximity of the uterine incision to the cervix, the formation of adhesions, myometrial ischemia, and impaired wound healing [8]. The technique used to close the uterus has evolved over the years [14]. In the UK, double-layer closure is recommended, with earlier studies suggesting that the effectiveness and safety of single-layer closure for the uterine incision remain uncertain (Nice guideline, [15]). In contrast, in several other countries, including the Netherlands and Belgium, the majority of gynecologists have shifted from double-layer to single-layer closure of the uterus.

Bujold et al. found in a multicenter case–control study that double-layer closure of the uterus halved the risk of uterine rupture in a future pregnancy compared with single-layer closure [16]. Yazıcıoğlu et al. examined two different uterine closure techniques after secondary and elective cesarean sections in a prospective cohort study of 78 patients [17]. This study found fewer isthmocele after single full-thickness uterine closure compared with split-thickness closure that excluded the endometrium.

Another theory regarding uterine closure involves locking and nonlocking sutures. Some studies suggest that the locking modification of a single-layer suture may increase the risk of uterine rupture due to increased tissue hypoxia and consequent impaired healing [18]. This issue has been explored in several studies. Yasmin et al. observed a reduction in myometrial thickness and an increase in blood loss with the use of first-layer locking [19]. In contrast, Ceci et al. found no significant difference in the occurrence of scar defects on comparing continuous, nonlocking, single-layer closure with interrupted, nonlocking, single-layer closure [20].

Recently, several authors have examined the association between two specific complications of cesarean delivery (CD), isthmocele formation and reduced residual myometrial thickness (RMT) at the scar site and their potential link to serious outcomes, such as uterine scar dehiscence and rupture in subsequent pregnancies. This hypothesis suggests that a decreased RMT may reflect a weaker uterine scar, thereby increasing the likelihood of uterine rupture or dehiscence in subsequent pregnancies [21–23].

An interesting observation is that most of the patients undergoing laparoscopic niche repair for large symptomatic isthmocele have a retroflexed uterus [5].

Recent studies have emphasized the importance of uterine closure with nonlocking continuous sutures. We planned this study with the idea that if we close the uterus in both layers without passing through the endometrium, we can increase the residual myometrial thickness and avoid isthmocele formation if upward retraction is prevented. In our study, we also examined the presence of isthmoceles and explored whether there is a correlation between uterine position and the development of isthmoceles by assessing uterine orientation.

The objectives of our study can be summarized as follows:

- 1. The effect of closure technique on isthmocele development
  This study aims to determine the effect of the closure technique on the development of isthmocele, considering the importance of the closure technique in preventing this condition.
- 2. Effect of suture technique on residual myometrial thickness (RMT)
  This outcome seeks to evaluate the effect of our suture technique on RMT.
- 3. The relationship between patient symptoms and the presence of isthmocele
  This outcome will investigate whether there is a relationship between patient symptoms and the presence of isthmocele.

## Materials and methods

**Fig. 1:**
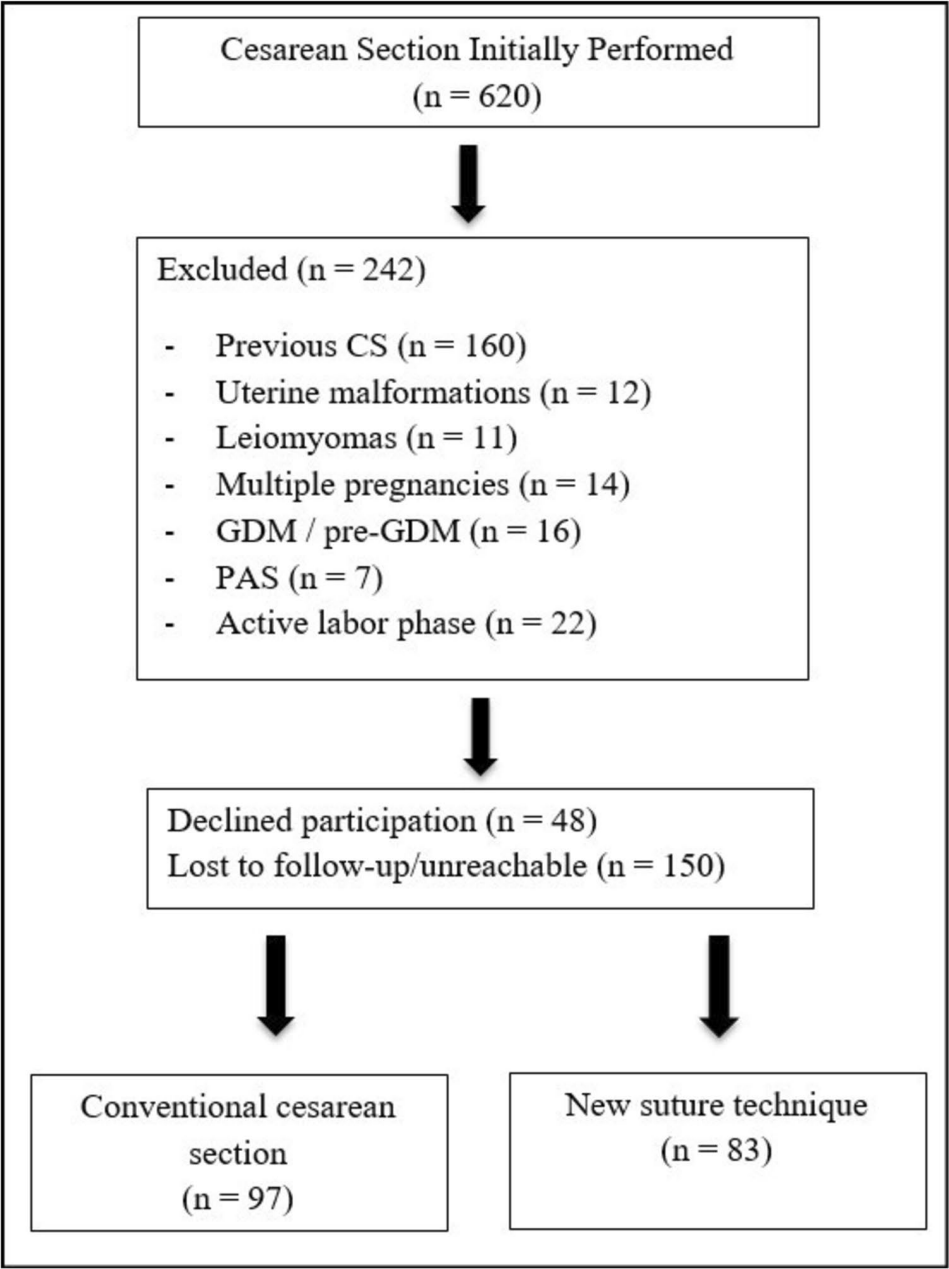
Study flow diagram comparing conventional and new suture techniques in cesarean delivery

This retrospective comparative cohort study was conducted at the Necmettin Erbakan University Teaching Hospital between March 2023 and January 2025, following approval from the local Ethics Committee. After a full explanation of the study’s objectives and procedures, low-segment uterine cesarean section was performed. The flow of patients included in the study and the group distribution are presented schematically. A total of 620 women were initially screened for eligibility. Of these, 378 met the predefined inclusion and exclusion criteria, and 180 women who attended the 12–24-week postpartum follow-up visit and underwent saline-infusion sonohysterography were finally included in the analysis (Fig. 1).

### Inclusion criteria

The study included patients undergoing elective or emergency cesarean section during the latent phase of labor. Eligible participants were primigravida and multigravida women aged 18–45 years with a singleton pregnancy. Cesarean delivery was performed in all viable pregnancies when indicated by cephalopelvic disproportion, fetal malpresentation (e.g., breech or transverse lie), placenta previa, fetal distress, fetal anomalies, or pregnancy complications such as preeclampsia.

### Exclusion criteria

Patients with previous cesarean section, uterine malformations, leiomyomas, PAS, previous isthmocele, a thin lower uterine segment, multiple pregnancies, macrosomia, and pregestational diabetes were not included in the study. Patients in the active phase of labor who were unable to deliver, as well as those with premature rupture of membranes, were excluded from the study. According to ACOG, the active phase of labor begins at 6 cm of cervical dilatation, which we adopted as the threshold for defining the active phase [24].

For the purpose of maintaining methodological consistency, a single experienced surgeon carried out all suturing procedures as well as all cesarean deliveries (JEH). Emergency cesarean sections performed by other surgeons were excluded from the study. Patients were retrospectively divided into two groups according to the uterine closure technique documented in the operative reports.

*Standard suture group* consisted of patients in whom the uterine incision was closed in double layer with locked suturation of the myometrium.

*New suture group* consisted of patients in whom the first layer of the uterine incision was closed continuously without locking and the second layer was closed with a ‘U’-shaped continuous suture.

### Low-segment uterine cesarean section (LSUCS)

A laparotomy was performed with a Pfannenstiel incision in the lower abdominal midline. The bladder was exposed through a peritoneal incision. A lower-segment transverse uterine incision was made approximately 1 cm below the peritoneal incision and then expanded. The membranes were cut, and both the baby and placenta were delivered. Cervical dilation was primarily assessed and managed using gloved finger dilation. In rare cases where this method was insufficient, Hegar bougie dilation was performed to prevent uterine hemorrhagic accumulation in patients with no cervical dilation. The suture material used had the same characteristics for both the case and control groups, with no differences in needle size or insertion intervals. For closure of the uterine myometrium layers, No. 1 Vicryl 50 mm suture [absorbable, multifilament, polyglycolic acid (PGA), and synthetic braided coating] was utilized. The visceral and parietal peritoneum was closed using a No. 2–0 26 mm [absorbable, multifilament, polyglycolic acid (PGA), and synthetic braided coating] suture. The criteria for placing ‘additional hemostatic sutures’ were quantitative: the presence of active or persistent leaking bleeding after the primary closure constituted an indication.

### Closure of the uterine incision for the standard suture group (double-layer, locked)

During the initial part of the study period, the senior surgeon (JEH) routinely used the conventional locked double-layer uterine closure technique. The unlocked double-layer, endometrium-sparing technique was subsequently introduced as the preferred method from March 2022 onward. In the standard suture group, the uterine incision was closed in two layers using a conventional locked technique. After the wound edges were gently approximated with forceps, a *Z*-stitch was placed at each corner of the incision. The width of the cervical canal was assessed, and Hegar dilatation was performed if necessary to ensure adequate drainage and prevent postoperative hematometra. The first layer of myometrial closure was performed using a continuous locked suture, taking full-thickness bites of the myometrium while carefully avoiding penetration of the endometrium or decidua. The second layer consisted of an additional continuous locked imbricating suture to reinforce the first layer and restore the normal uterine contour. The parietal peritoneum and visceral peritoneum were then closed in accordance with standard surgical practice. The two techniques were not used in parallel. The traditional locked technique was applied until March 2022, after which the unlocked double-layer, endometrium-protective technique was adopted.

### Closure of the uterine incision for the new suture group (double-layer, unlocked, U shapes)

**Fig. 2:**
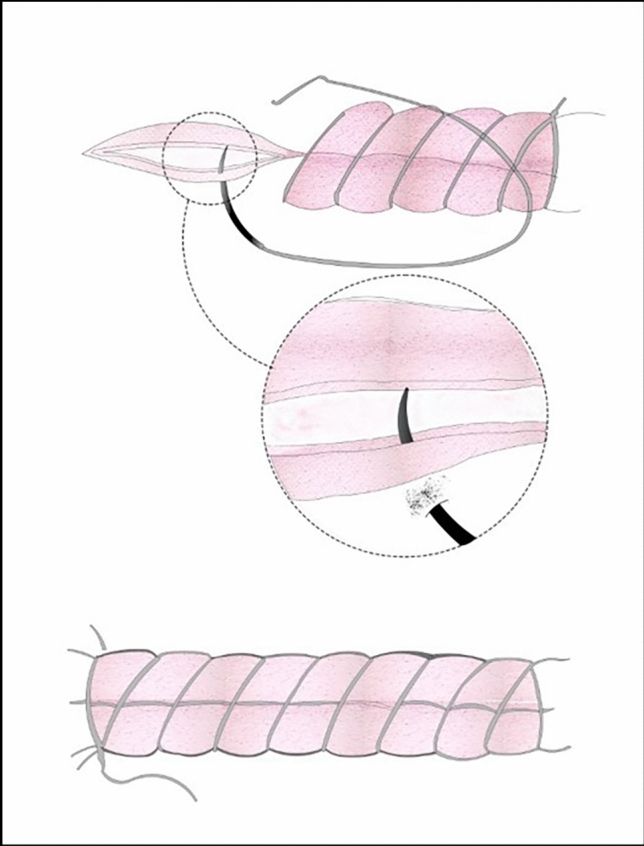
Continuous unlocked first-layer closure of the uterine incision without endometrial involvement

**Fig. 3:**
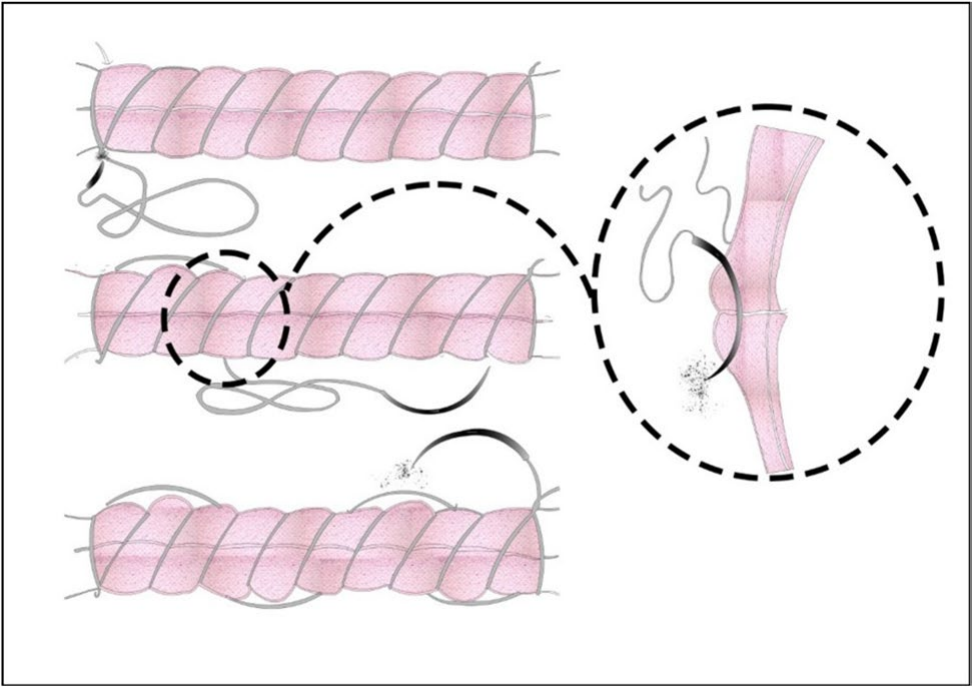
U-shaped continuous second-layer closure confined to the myometrium

The wound edges were gently approximated using mucosal forceps, and a *Z*-stitch was placed at each corner of the incision. The width of the cervical canal was assessed, and Hegar bougie dilatation was performed when necessary. The first layer of the uterine incision was closed using a continuous unlocked suture, carefully avoiding penetration of the endometrium (Fig. 2). The second layer, consisting of the remaining outer half of the myometrium, was reinforced with continuous *U*-shaped sutures to restore the normal uterine contour (Fig. 3). Both the parietal and visceral peritoneum were then closed according to standard surgical practice.

The presence of an isthmocele was evaluated in all patients using saline-infusion sonohysterography (SIS). In accordance with the modified Delphi consensus, an isthmocele was defined as an anechoic defect within the myometrium of the lower uterine segment measuring at least 2.0 mm in depth [2].

### Saline infusion sonohysterography (SIS)

Under aseptic precautions, a sterile Sims speculum was inserted. The intrauterine catheter was advanced from the external cervical os into the endometrial cavity. The speculum was then removed, and the endovaginal probe was positioned next to the catheter. Under direct sonographic guidance, the balloon was gently withdrawn to occlude the internal cervical os, and 15–20 mL of saline was injected into the endometrial cavity to separate the opposing walls of the endometrium. The area of the previous cesarean section and the endometrium were visualized. The presence of an isthmocele was assessed, and the residual myometrial thickness was measured. Residual myometrial thickness (RMT) was measured during saline-infusion sonohysterography in the sagittal plane at the level of the previous cesarean scar. Measurements were obtained perpendicular to the endometrial cavity using electronic calipers placed from the deepest point of the niche (or scar indentation) to the serosal surface of the uterus, representing the minimal remaining myometrial layer. All measurements were performed under real-time transvaginal ultrasound guidance using a standardized measurement protocol by the senior author (J.E.H.). Interobserver variability was not assessed, because measurements were obtained by a single assessor.

### Data collection

Patients in the standard suture group and the new suture group were called for a follow-up visit between 12 and 24 weeks after their cesarean section surgery. During these follow-ups, the patients’ sociodemographic data and symptoms (such as postmenstrual spotting, dysmenorrhea, and pelvic pain) were evaluated. Symptom assessment (including postmenstrual spotting, dysmenorrhea, and chronic pelvic pain) was performed during the 12–24-week postpartum follow-up visit, concurrently with the SIS examination. Patients underwent saline-infusion sonography via transvaginal ultrasonography to evaluate the presence of isthmocele and residual myometrial thickness (RMT). All data were recorded, and statistical comparisons were made between the two groups. Because this was a retrospective cohort study, participants were not randomized. Allocation to the standard or new suture group was determined solely by the uterine closure technique recorded in the operative notes, reflecting the surgeon’s routine clinical practice at the time of surgery. Preoperative anemia was defined as a hemoglobin level < 11.0 g/dL.

All women who underwent low-segment uterine cesarean section performed by the senior author (JEH) at Necmettin Erbakan university teaching hospital between March 2023 and January 2025 were screened for eligibility according to the predefined inclusion and exclusion criteria. Potentially eligible women were identified from the institutional electronic obstetric database and operating theater logbooks during the early postpartum period. Beginning approximately 6 weeks after delivery, these women were contacted by telephone and invited to attend a dedicated outpatient visit scheduled between 12 and 24 weeks postpartum. At this visit, the study protocol was explained in detail, written informed consent was obtained, and saline-infusion sonohysterography (SIS) was performed. Women who declined to participate, who did not wish to undergo additional imaging, who could not be reached, or who failed to attend the scheduled appointment were not enrolled and were, therefore, not included in the final analysis.

### Statistical analysis

All statistical analyses were performed using IBM SPSS Statistics for Windows, Version 26.0 (IBM Corp., Armonk, NY, USA). The normality of distribution for continuous variables was assessed using the Shapiro–Wilk test and histograms. Continuous variables with a normal distribution were presented as mean ± standard deviation (SD), while those not normally distributed were expressed as median (minimum–maximum). Categorical variables were reported as numbers and percentages (%). For comparisons between groups, the independent samples *t *test was used for normally distributed continuous variables, and the Mann–Whitney *U* test was applied for non-normally distributed variables. Categorical variables were compared using the Chi-square test or Fisher’s exact test, as appropriate. To identify independent predictors of isthmocele development, a multivariable binary logistic regression analysis was performed. The model included the following variables: age, BMI, gravidity, parity, gestational age at delivery, birth weight, cervical dilatation, operation time, preoperative anemia, uterine position, and suture technique group. To investigate the factors influencing residual myometrial thickness, a multiple linear regression analysis was conducted. Multicollinearity was assessed using VIF and tolerance values, and no concerning multicollinearity was detected (all VIF < 2). A two-tailed *p *value of < 0.05 was considered statistically significant for all tests.

## Results

### Patient demographics and maternal characteristics

A total of 180 patients were included in the study, with 97 undergoing standard suture group and 83 receiving the new suture technique. As shown in Table 1, there were no significant differences between the two groups in terms of age, body mass index (BMI), gravidity, parity, and number of abortions. No statistically significant differences in baseline demographics were observed between the two groups. Of the 378 eligible women invited for follow-up, 150 did not attend the scheduled 12–24-week postpartum evaluation, corresponding to a loss to follow-up rate of 39.7%. Additionally, the basic demographic and obstetric characteristics of women who participated in SIS monitoring were compared with those who did not. As shown in Supplementary Table (Table 1), no significant differences were observed between the groups in terms of age, body mass index, number of pregnancies, number of live births, gestational age at delivery, birth weight, rates of preoperative anemia, and reasons for cesarean section (all *p* > 0.05).

**Table 1:** Comparison of demographic and maternal characteristics between conventional cesarean section and new suture technique groups

Variables	Conventional cesarean section (*n* = 97)	New suture technique (*n* = 83)	*p *value
Age (years)	30.61 ± 5.41	31.08 ± 4.85	0.547
BMI (kg/m^2^)	27.76 ± 4.97	28.57 ± 4.91	0.279
Gravidity	3 (1–5)	3 (1–6)	0.265
Parity	2 (1–5)	2 (1–6)	0.269
Abortion	0 (0–3)	0 (0–4)	0.985

*BMI* body mass index

### Intraoperative and postoperative outcomes

Table 2 summarizes the intraoperative and postoperative findings. The new suture technique group demonstrated a significantly shorter operation time (median 26 min vs. 33 min for conventional, *p* = 0.001). While preoperative and postoperative hemoglobin levels were statistically higher in the new suture group (*p* = 0.003 and *p* = 0.001, respectively), the clinical significance of these differences was deemed negligible, and the change in hemoglobin levels between the groups was not statistically significant (*p* = 0.124). The incidence of preoperative anemia was comparable between both groups (27.8% vs. 22.9%, *p* = 0.448). A notable finding was the significantly reduced need for additional hemostatic sutures in the new suture group (2.4% vs. 11.3%, OR = 5.18 (conventional vs new), 95%CI 1.11 − 24.1, *p* = 0.021). Gestational age at delivery, birth weight, cervical dilatation, and cesarean indications did not show significant differences between the two techniques.

**Table 2:** Comparison of intraoperative and postoperative outcomes between conventional cesarean section and new suture technique groups

Variables	Conventional cesarean section (*n* = 97)	New suture technique (*n* = 83)	*p *value
Cervical dilatation (cm)	0 (0–4)	0 (0–3)	0.971
Operation time (min)	33 (25–45)	26 (20–30)	0.001
Preoperative Hgb (g/dL)	11.47 ± 0.81	11.84 ± 0.85	0.003
Postoperative Hgb (g/dL)	10.31 ± 0.86	10.78 ± 0.71	0.001
Change of Hgb (g/dL)	1.15 ± 0.49	1.06 ± 0.36	0.124
Preoperative anemia	27 (27.8%)	19 (22.9%)	0.448
Need for additional hemostatic sutures	11 (11.3%)	2 (2.4%)	0.021 OR = 5.18 (95%CI 1.11–24.1)
Gestational age at delivery (weeks)	38 (31–40)	38 (34–40)	0.872
Birth weight (g)	3238.40 (1160–4000)	3290 (1947.15–4000)	0.710
*C/S indications*			
Fetal distress	32(33.0%)	24 (28.9%)	0.677
Breech/abnormal presentation	23 (23.7%)	23 (27.7%)	
FGR	16 (16.5%)	11 (13.3%)	
Preeclampsia	12 (12.4%)	10 (12.0%)	
Oligohydramnios	13 (13.4%)	11 (13.3%)	
Placenta previa	1 (1.0%)	4 (4.8%)	

*C/S* cesarean section, *FGR* fetal growth restriction

### Uterine healing outcomes and symptoms

As presented in Table 3, the new suture technique significantly improved uterine healing and reduced related symptoms. The residual myometrial thickness was significantly greater in the new suture group (13.91 ± 5.44 mm vs. 10.18 ± 5.13 mm, *p* = 0.001). Furthermore, residual myometrial thickness less than 5 mm was exclusively observed in the standard suture group (18.6% vs. 0%, *p* = 0.001), highlighting superior anatomical integrity with the new technique. An RMT of less than 5 mm was only observed in the standard suture group. An additional analysis considering lower threshold values determined that RMT never fell below 2.5 mm during the study, with the minimum measured value being 2.5 mm. The incidence of isthmocele was substantially lower in the new suture group (7.2% vs. 39.2%, OR = 8.27, 95%CI 3.28–20.85, *p* = 0.001). Likewise, the rates of postmenstrual spotting (8.4% vs. 43.3%, OR = 8.29, 95%CI 3.46–19.83, *p* = 0.001), dysmenorrhea (15.7% vs. 57.7%, OR = 7.35, 95%CI 3.59–15.05, *p* = 0.001), and chronic pelvic pain (9.6% vs. 48.5%, OR = 8.81, 95%CI 3.84–20.22, *p* = 0.001) were all significantly reduced in the new suture group. Uterine position (retroversion vs. anteversion) did not differ significantly between the groups (*p* = 0.196).

**Table 3:** Comparison of uterine healing outcomes and symptoms between conventional cesarean section and new suture technique groups

Variables	Conventional cesarean section (*n* = 97)	New suture technique (*n* = 83)	OR (95%CI)	*p *value
RMT (mm)	10.18 ± 5.13	13.91 ± 5.44		0.001
RMT < 5 mm	18 (18.6%)	0 (0.0%)		0.001
Isthmocele	38 (39.2%)	6 (7.2%)	8.27 (3.28–20.85)	0.001
Spotting after menstruation	42 (43.3%)	7 (8.4%)	8.29 (3.46–19.83)	0.001
Dysmenorrhea	56 (57.7%)	13 (15.7%)	7.35 (3.59–15.05)	0.001
Chronic pelvic pain	47 (48.5%)	8 (9.6%)	8.81 (3.84–20.22)	0.001
*Uterine position*				
Retroversion	8 (8.2%)	3 (3.6%)		0.196
Anteversion	89 (91.8%)	80 (96.4%)		

*RMT* residual myometrial thickness

### Predictors of isthmocele formation and residual myometrial thickness

Logistic regression analyses have demonstrated a consistent association between the uterine closure technique used and the formation of isthmoceles. In the crude (univariate) analysis, the traditional locked closure method was found to be significantly more likely to result in isthmocele formation than the unlocked double-layer technique (crude odds ratio (OR) = 0.121, 95% confidence interval (CI) 0.048–0.305; *p* < 0.001). However, after adjusting for maternal demographic factors (age, BMI, and number of pregnancies and births), obstetric characteristics (gestational age at delivery, birth weight and cervical dilation), and surgical variables (surgery duration, preoperative anemia, and uterine position), the nonlocked technique was found to be the only independent predictor of isthmocele formation (adjusted OR = 0.059, 95%CI 0.018–0.198; *p* < 0.001). None of the examined covariates, including parity, BMI, or preoperative anemia, were found to be statistically significant in the adjusted model (Table 4).

**Table 4:** Binary logistic regression analysis for predicting isthmocele formation

Predictor	Crude or (95%CI)	*p *value	Adjusted or (95%CI)	*p *value
Group (conventional vs new)	8.27 (3.28–20.85)	0.001	15.77 (5.12–48.55)	0.001
Age (years)	1.01 (0.94–1.08)	0.847	1.07 (0.99–1.16)	0.112
BMI (kg/m^2^)	1.04 (0.97–1.11)	0.302	1.08 (0.99–1.17)	0.096
Gravidity	1.01 (0.75–1.37)	0.948	1.05 (0.63–1.75)	0.842
Parity	0.89 (0.65–1.22)	0.478	0.73 (0.43–1.23)	0.236
Gestational age (weeks)	1.15 (0.92–1.44)	0.211	1.29 (0.72–2.33)	0.389
Birth weight (g)	1.00 (0.9996–1.0004)	0.382	1.00 (0.998–1.002)	0.782
Cervical dilatation (cm)	1.30 (0.91–1.85)	0.155	1.30 (0.84–2.02)	0.246
Operation time (min)	1.04 (0.97–1.12)	0.242	0.92 (0.83–1.02)	0.115
Preoperative anemia (yes vs no)	2.02 (0.97–4.22)	0.061	2.06 (0.83–5.12)	0.121
Uterine retroversion (vs anteversion)	1.84 (0.51–6.62)	0.349	1.41 (0.32–6.14)	0.649

*BMI* body mass index

Multiple linear regression analysis (Table 5) on residual myometrial thickness showed that the new suture technique (*β* = 0.492, *p* = 0.001) and increased parity (*β* = 0.210, *p* = 0.035) were significantly associated with greater thickness. Preoperative anemia was also negatively associated with residual myometrial thickness (*β* = –0.212, *p* = 0.003), suggesting a potential impact of hematologic status on healing. Operation time and BMI were not statistically significant predictors in the updated model (*p* = 0.149 and *p* = 0.151, respectively).

**Table 5:** Multiple linear regression analysis for predictors of residual myometrial thickness

Predictor	*B*	Std. error	*β* (beta)	*t*	*p *value	95%CI for *B* (lower–upper)
(Constant)	13.127	15.548	–	0.844	0.400	− 17.568 to 43.823
Age (years)	0.087	0.069	0.087	1.260	0.209	− 0.049 to 0.222
BMI (kg/m^2^)	0.098	0.068	− 0.096	− 1.442	0.151	− 0.233 to 0.036
Gravidity	− 0.520	0.453	− 0.115	− 1.149	0.252	− 1.415 to 0.374
Parity	0.951	0.449	0.210	2.120	0.035	0.065 to 1.837
Gestational age at delivery (weeks)	− 0.202	0.522	− 0.064	− 0.388	0.699	− 1.233 to 0.828
Birth weight (g)	0.004	0.002	0.005	0.029	0.977	− 0.003 to 0.003
Cervical dilatation (cm)	− 0.426	0.399	− 0.074	− 1.069	0.287	− 1.213 to 0.361
Operation time (min)	0.134	0.093	0.136	1.451	0.149	− 0.048 to 0.317
Group (conventional vs new)	5.019	0.935	0.492	5.367	0.001	3.173 to 6.866
Preoperative anemia	− 2.475	0.823	− 0.212	− 3.008	0.003	− 4.099 to − 0.851
Uterine retroversion	− 1.777	1.400	− 0.084	− 1.270	0.206	− 4.540 to 0.986

*BMI* body mass index

## Discussion

This study provides preliminary evidence for the novel unlocked double-layer uterine closure technique in cesarean delivery, particularly regarding uterine healing and the prevention of isthmocele-related complications. The new method, conversely, resulted in significantly increased residual myometrial thickness (RMT), a reduced incidence of isthmocele, and fewer occurrences of spotting, dysmenorrhea, and chronic pelvic pain postmenstruation. These findings are significant due to the global increase in cesarean delivery rates and the growing awareness of potential long-term complications, such as niche formation, associated with cesarean sections. Although the two groups demonstrated broadly comparable baseline demographic and maternal characteristics, this similarity can only reduce—but not fully eliminate—the possibility of residual confounding inherent to retrospective observational designs.

The new suture group shorter operative time (median 26 vs. 33 min) and noticeably lower need for additional hemostatic suturing were two more clinically significant intraoperative findings. Although the new technique group's hemoglobin levels were marginally better preserved, the differences were not considered clinically significant. However, these findings imply that the new approach might be more efficient in addition to being more effective.

Studies that came before this one have shown different results about how uterine closure methods affect the formation of isthmocele. For example, a meta-analysis [25] and a prospective cohort study by Osser and Valentin [26] found that there was no significant difference in the number of isthmocele cases between single-layer and double-layer uterine closure methods. Most previous studies compared single-layer vs. double-layer closure. Our study specifically addressed the impact of locking vs. unlocking within the double-layer technique.

Randomized-controlled trials provide important yet heterogeneous evidence regarding the optimal uterine closure technique. Roberge et al. demonstrated that unlocked double-layer closure resulted in significantly greater residual myometrial thickness and improved healing compared with single-layer closure, whereas locked double-layer closure did not confer the same advantage [27]. Similarly, Bennich et al. reported that adding a second unlocked layer increased RMT without increasing operative morbidity [23]. In contrast, Osser and Valentin found no significant differences in niche formation or clinical symptoms between certain single- and double-layer approaches in their randomized comparison, suggesting that the influence of closure technique may vary across patient populations and surgical conditions [26]. More recent analyses, including the systematic review by Marchand et al., indicate that while unlocked and endometrium-sparing techniques may improve anatomical healing, the overall strength of evidence remains mixed and highlights the need for large, standardized trials [28]. Taken together, existing RCTs reinforce that suture locking, endometrial involvement, and the number of layers may each contribute to uterine scar healing, but definitive conclusions remain limited by study heterogeneity.

These results suggest that the way the closure is done may not be a major factor in the development of isthmocele. However, other studies have suggested that double-layer closure, especially methods that do not damage the endometrium, can lower the risk of isthmocele formation [29]. These differences show that we need more well-planned, large-scale prospective studies to better understand how suturing techniques can help stop isthmocele. When suturing, the uterine wound may completely fold, which could cause the endometrial lining to stay in the myometrium and possibly help isthmocele grow. Some studies suggest that passing the needle through the myometrial-endometrial junction and using a suturing technique that does not involve the endometrium [30] can help the compartments of the uterus line up better and speed up healing. In our study, we applied double-layer suture to all patients without passing through the endometrium and compared the locked and nonlocked sutures.

One of the most interesting things we found in our study was that the new suture group had a much lower rate of isthmocele (7.2%) than the standard suture group (39.2%), with an odds ratio of 8.27. This big difference is in line with what Alper et al. [31] found; parallel layer closure not only led to fewer isthmocele formations, but it also left a thicker layer of myometrial tissue behind than single-layer closure in women who had a primary cesarean section. Their study also showed that patients in the single-layer closure group had more symptoms related to isthmocele, such as spotting after their period and dysmenorrhea. Roberge et al. also stressed the importance of opening the first suture layer and leaving the endometrium alone, as these methods led to better healing of the myometrium and less niche-related morbidity. These results support the idea that both the number of layers and the specific characteristics of the suturing method—especially avoiding locking and penetrating the endometrium—are very important for getting the best healing of uterine scars and reducing long-term gynecologic symptoms [31]. A broader literature on wound closure supports the view that the closure strategy itself is a modifiable factor that determines the quality of healing. Libretti and colleagues recently demonstrated that a particular closure technique can reduce operative time and wound complications while preserving cosmetic outcomes in gynecological surgery [32]. While their work focused primarily on abdominal and cutaneous closure rather than hysterotomy repair, the underlying principle is consistent with our findings: the technical details of closure, such as locking and unlocking, and the preservation of tissue planes can significantly influence scar dynamics. Our results show that applying this principle at the myometrial level and using an unlocked double-layer approach can promote anatomical healing after cesarean section. The anatomical superiority of the new technique was further demonstrated by our study, which found that patients who underwent the standard suture were the only ones with residual myometrial thickness less than 5 mm.

In another review comparing double-layer uterine closure to single-layer closure, residual myometrial thickness was also found to be greater in the double-layer suturing group; however, no significant difference was observed in the incidence of isthmocele formation [28]. Similarly, Roberge et al. found that, among 73 participants, double-layer closure with an unlocked first layer resulted in a thicker RMT and a higher healing rate compared to single-layer closure. In contrast, double-layer closure with a locked first layer did not show a significant difference in RMT or healing rate when compared to single-layer closure [27]. Consistent with these findings, our study also demonstrated that using a double-layer, unlocked suture technique where the first layer was continuous and nonlocking and the second was applied in a *U*-shaped fashion led to significantly greater RMT and a markedly lower incidence of isthmocele compared to the standard suture double-layer locked method. These results support the growing body of evidence, suggesting that not only the number of layers but also the nature of the suturing (locked vs. unlocked) plays a critical role in uterine scar healing and the prevention of isthmocele formation. In our study, the fact that RMT did not fall below 2.5 mm suggests that a certain baseline level of uterine scar healing was maintained in both groups from an anatomical perspective. However, if supported by prospective studies that include subsequent pregnancy outcomes, our findings may become more important as higher-quality studies emerge that help us better understand the clinical significance of RMT.

Significantly lower rates of postmenstrual spotting (8.4% vs. 43.3%), dysmenorrhea (15.7% vs. 57.7%), and chronic pelvic pain (9.6% vs. 48.5%) were among the symptoms that patients who received the new suture technique reported. These results have clinical significance, since they are directly related to decreased gynecologic morbidity and enhanced postoperative quality of life. It is commonly known that abnormal uterine bleeding and persistent pelvic symptoms, especially in women of reproductive age, are linked to thin residual myometrium and niche formation [6, 7, 12]. These findings are consistent with new research showing a strong correlation between the shape of uterine scar tissue and symptom severity. In a study of women who had previously undergone cesarean sections, Kellner et al. (2024) found that smaller amounts of residual myometrium and specific niche morphology were strongly associated with postmenstrual spotting, painful periods, and persistent pelvic pain. These results reinforce our conclusion that fewer clinical complaints in the unblocked group are linked to higher levels of residual myometrium (RMT) [33].

Logistic regression analysis showed that the suture technique was the only independent predictor of isthmocele formation (OR = 0.059, 95%CI 0.018–0.198, *p* = 0.001). This means that the surgical approach is more important for healing uterine scars than factors related to the patient, such as age, parity, BMI, or gestational factors. This odds ratio shows that using the new suture technique made it 94.1% less likely that someone would get an isthmocele than using the old method. This big protective effect shows how important the unlocked double-layer, endometrium-sparing closure technique is in the clinic.

At the same time, our multiple linear regression analysis showed that both higher parity and the use of the new suture technique were linked to higher RMT levels on their own, which shows how they help the uterus heal. In contrast, anemia before surgery was found to be a strong negative predictor of RMT, which means that it hurts the body's ability to heal after surgery. There is already research that backs up this finding. It has shown that anemia slows down the healing of wounds by reducing the amount of oxygen that gets to tissues that are healing and changing the way collagen is made [34]. Specifically, it has been demonstrated that anemia increases vulnerability to scar-related complications in the postpartum phase and delays uterine involution [34]. Although group-level differences in hemoglobin were not clinically significant, regression analysis revealed that preoperative anemia independently predicted poorer healing (lower RMT). Although group-level Hb differences were clinically negligible, preoperative anemia as a physiological variable was independently associated with impaired healing. Therefore, maximizing uterine recovery following caesarian section requires the detection and efficient treatment of maternal anemia. All things considered, these results demonstrate how systemic physiological conditions like hematological status and mechanical factors like suture technique interact to influence the quality of uterine scar formation. They also stress that surgical technique is a modifiable factor that can greatly improve caesarian outcomes, especially when paired with careful perioperative hematological management.

The clinical significance of RMT has been increasingly emphasized in recent literature. Several studies have suggested that thinner residual myometrial tissue—commonly defined as < 2.5–3.0 mm—may be associated with an elevated risk of uterine scar dehiscence, abnormal uterine bleeding, and niche-related symptoms in the nonpregnant state [26]. Moreover, an RMT below 2.5 mm has been proposed as a potential risk factor for uterine rupture or severe scar defects in subsequent pregnancies, particularly when combined with a deeply embedded niche [22]. Even moderate thinning (< 5 mm) has been associated with impaired uterine integrity and poorer symptom profiles [6, 9]. In this context, the significantly greater residual myometrial thickness (RMT) observed in the novel suture group should be interpreted primarily as an anatomical indicator of improved uterine scar healing. While such findings may suggest potential long-term reproductive benefits, it is important to note that our study does not include data on subsequent pregnancy outcomes. Therefore, any obstetric implications remain speculative and require confirmation through prospective studies involving pregnancy follow-up [23].

The clinical implications of these findings merit consideration [7]. These findings are consistent with our recent cross-sectional analysis, which also demonstrated a cumulative increase in isthmocele prevalence with repeated cesarean deliveries [35]. A thicker residual myometrial layer and a markedly lower incidence of isthmocele may translate into fewer long-term gynecologic symptoms, particularly abnormal uterine bleeding, postmenstrual spotting, and chronic pelvic pain [6, 9]. Improved myometrial integrity may also have relevance for future reproductive outcomes, as thinner scars and deep niches have been associated with impaired fertility and increased risks in subsequent pregnancies. Although our study was not designed to evaluate obstetric outcomes, a more robust uterine scar may theoretically reduce the risk of uterine dehiscence or rupture during labor and may influence counseling regarding VBAC eligibility [21]. These potential benefits underscore the need for well-designed prospective and multicenter studies to determine whether the anatomical advantages observed with the novel technique translate into measurable improvements in long-term reproductive and obstetric outcomes.

### Strengths of the study

This study also has several notable strengths. First, all cesarean procedures were performed by a single experienced surgeon, thereby minimizing variability in operative technique and ensuring consistency in suture application. Second, the same type and size of suture material were used in all cases, reducing potential material-related heterogeneity. Third, postoperative evaluation of isthmocele and residual myometrial thickness was performed uniformly using SIS, providing a standardized and sensitive assessment of uterine healing. Finally, the use of multivariable regression models allowed adjustment for several potential confounders, strengthening the internal validity of the observed associations.

## Limitations

There are several important limitations to this study. Additionally, the study was nonrandomized, and allocation to the suture groups depended on the surgeon’s routine technique rather than controlled assignment, which may introduce selection bias. First, although the two groups were broadly comparable, the retrospective cohort design inherently limits the ability to draw causal inferences, and residual confounding cannot be fully excluded. Second, all procedures were performed by a single experienced surgeon, which ensured technical consistency but limits the generalizability of the findings to wider clinical practice. Third, emergency cesarean sections performed by other surgeons were intentionally excluded to maintain methodological standardization; however, this restriction reduces the applicability of the results to predominantly elective or semi-elective cases. Fourth, uterine healing was assessed at a single postpartum time point (12–24 weeks), which may not fully capture the dynamic and prolonged process of scar remodeling. Fifth, the substantial loss to follow-up (39.7%) may introduce selection bias and should be considered when interpreting the results. Sixth, although multivariable models were used, residual confounding related to preoperative anemia or other unmeasured obstetric factors cannot be completely excluded. Seventh, the sample size, although adequate for detecting the main effect, remains modest and may have limited the ability to identify smaller associations. Eighth, as the transition to the new closure technique occurred at a specific point in time, it was not possible to separate the effect of the surgical year variable during the study period. Consequently, the surgical year could not be included in the model, meaning that the impact of potential changes in surgical practices or perioperative care over time could not be fully eliminated. Ninth, the high follow-up loss rate of 39.7% increases the risk of selection bias in the study. Although no significant differences were observed between SIS participants and nonparticipants in terms of basic clinical and demographic variables (see Supplementary Table 1), this may limit the generalizability of our results to the entire cesarean population. Finally, key obstetric outcomes—such as future fertility, uterine rupture, and the success of vaginal birth after cesarean section (VBAC)—were not assessed, and therefore, the long-term predictive value of residual myometrial thickness remains uncertain. A significant limitation of this study is that the two uterine closure techniques were not applied in parallel. Consequently, the differences observed between the groups may stem from temporal effects, such as perioperative management, surgical experience, hemostatic practices, documentation quality, or patient variability over time, rather than the suture technique itself.

An important limitation is that outcomes were assessed only in women who returned for follow-up SIS. If symptoms influenced follow-up attendance, selection bias may have occurred. Although no significant differences were found in measured variables between those who attended and those who did not, unmeasured factors related to differential follow-up may have influenced the estimated rates of isthmocele and related symptoms.

Uterine scar remodeling is a time-dependent process, and the appearance of a niche as well as residual myometrial thickness may change over the early postpartum period. In this study, SIS was performed between 12 and 24 weeks after delivery, and variability in imaging timing may have affected niche detection and thickness measurements. Because the two closure techniques were implemented sequentially, systematic differences in follow-up timing between groups cannot be excluded and may have introduced measurement bias. Future studies should standardize the timing of postoperative imaging or perform stratified analyses according to postpartum interval.

## Conclusion

In conclusion, this retrospective cohort study suggests that the unlocked, double-layer, endometrium-sparing uterine closure technique may be associated with improved short-term uterine healing, greater residual myometrial thickness, and lower rates of isthmocele-related symptoms compared with the conventional locked technique. However, these findings should be interpreted with caution given the study’s methodological limitations, including its retrospective design, single-surgeon cohort, loss to follow-up, and lack of long-term obstetric outcomes. Larger, prospective, and multicenter studies are needed to validate these preliminary observations and to determine whether the anatomical advantages observed translate into improved reproductive and obstetric outcomes. This work lays the groundwork for future research to determine whether these anatomical differences translate into measurable reproductive or obstetric benefits.

## Supplementary Information

Below is the link to the electronic supplementary material.

Supplementary file1 (MP4 8653 KB)

Supplementary file2 (DOCX 15 KB)

## Data Availability

The datasets generated and/or analyzed during the current study are not publicly available but are available from the corresponding author on reasonable request.

## References

[1] Boerma T, Ronsmans C, Melesse DY, Barros AJ, Barros FC, Juan L et al (2018) Global epidemiology of use of and disparities in caesarean sections. Lancet 392(10155):1341–1348 30322584 10.1016/S0140-6736(18)31928-7

[2] Jordans I, De Leeuw R, Stegwee S, Amso N, Barri-Soldevila P, Van Den Bosch T et al (2019) Sonographic examination of uterine niche in non-pregnant women: a modified Delphi procedure. Ultrasound Obstet Gynecol 53(1):107–115 29536581 10.1002/uog.19049 PMC6590297

[3] Monteagudo A, Carreno C, Timor-Tritsch IE (2001) Saline infusion sonohysterography in nonpregnant women with previous cesarean delivery: the" niche" in the scar. J Ultrasound Med 20(10):1105–1115 11587017 10.7863/jum.2001.20.10.1105

[4] Morris H (1995) Surgical pathology of the lower uterine segment caesarean section scar: is the scar a source of clinical symptoms? Int J Gynecol Pathol 14(1):16–20 7883420 10.1097/00004347-199501000-00004

[5] Bij de Vaate A, Van der Voet L, Naji O, Witmer M, Veersema S, Brölmann H et al (2014) Prevalence, potential risk factors for development and symptoms related to the presence of uterine niches following cesarean section: systematic review. Ultrasound in Obstet Gynecol 43(4):372 10.1002/uog.13199 23996650

[6] van der Voet L, de Bij Vaate A, Veersema S, Brölmann H, Huirne J (2014) Long-term complications of caesarean section. The niche in the scar: a prospective cohort study on niche prevalence and its relation to abnormal uterine bleeding. BJOG: Int J Obstet and Gynaecol 121(2):236–244 10.1111/1471-0528.12542 24373597

[7] Tulandi T, Cohen A (2016) Emerging manifestations of cesarean scar defect in reproductive-aged women. J Minim Invasive Gynecol 23(6):893–902 27393285 10.1016/j.jmig.2016.06.020

[8] Vervoort A, Uittenbogaard L, Hehenkamp W, Brölmann H, Mol B, Huirne J (2015) Why do niches develop in Caesarean uterine scars? Hypotheses on the aetiology of niche development. Hum Reprod 30(12):2695–2702 26409016 10.1093/humrep/dev240 PMC4643529

[9] de Bij Vaate A, Brölmann H, Van Der Voet L, Van Der Slikke J, Veersema S, Huirne J (2011) Ultrasound evaluation of the cesarean scar: relation between a niche and postmenstrual spotting. Ultrasound Obstet Gynecol 37(1):93–99 21031351 10.1002/uog.8864

[10] Thurmond AS, Harvey WJ, Smith SA (1999) Cesarean section scar as a cause of abnormal vaginal bleeding: diagnosis by sonohysterography. J Ultrasound Med 18(1):13–16 9952074 10.7863/jum.1999.18.1.13

[11] Ash A, Smith A, Maxwell D (2007) Caesarean scar pregnancy. BJOG: An Int J Obstet Gynaecol 114(3):253–263 10.1111/j.1471-0528.2006.01237.x 17313383

[12] Raimondo G, Grifone G, Raimondo D, Seracchioli R, Scambia G, Masciullo V (2015) Hysteroscopic treatment of symptomatic cesarean-induced isthmocele: a prospective study. J Minim Invasive Gynecol 22(2):297–301 25285773 10.1016/j.jmig.2014.09.011

[13] Dominoni M, Torella M, Molitierno R, Fordellone M, Saccone G, Colacurci D et al (2025) Single-versus double-layer uterine closure at the time of cesarean delivery and risk of uterine scar niche: a systematic review and meta-analysis of randomized trials. Arch Gynecol Obstet 312(4):1095–1106 40833607 10.1007/s00404-025-08151-y PMC12414086

[14] Pandit SN, Khan RJ (2013) Surgical techniques for performing caesarean section including CS at full dilatation. Best Pract Res Clin Obstet Gynaecol 27(2):179–195 23415448 10.1016/j.bpobgyn.2012.12.006

[15] Cohen MA, Chen CCG (2021) Evidence based cesarean section. Glob Libr Women’s Med. 10.3843/GLOWM.415553

[16] Bujold E, Goyet M, Marcoux S, Brassard N, Cormier B, Hamilton E et al (2010) The role of uterine closure in the risk of uterine rupture. Obstet Gynecol 116(1):43–50 20567166 10.1097/AOG.0b013e3181e41be3

[17] Yazicioglu F, Gökdogan A, Kelekci S, Aygün M, Savan K (2006) Incomplete healing of the uterine incision after caesarean section: is it preventable? Eur J Obstet Gynecol Reprod Biol 124(1):32–36 16023780 10.1016/j.ejogrb.2005.03.023

[18] Roberge S, Chaillet N, Boutin A, Moore L, Jastrow N, Brassard N et al (2011) Single-versus double-layer closure of the hysterotomy incision during cesarean delivery and risk of uterine rupture. Int J Gynaecol Obstet 115(1):5–10 21794864 10.1016/j.ijgo.2011.04.013

[19] Yasmin S, Sadaf J, Fatima N (2011) Impact of methods for uterine incision closure on repeat caesarean section scar of lower uterine segment. J Coll Physicians Surg Pak 21(9):522–526 21914406

[20] Ceci O, Cantatore C, Scioscia M, Nardelli C, Ravi M, Vimercati A et al (2012) Ultrasonographic and hysteroscopic outcomes of uterine scar healing after cesarean section: comparison of two types of single‐layer suture. J Obstet Gynaecol Res 38(11):1302–1307 22612785 10.1111/j.1447-0756.2012.01872.x

[21] Rozenberg P, Goffinet F, Philippe H, Nisand I (1996) Ultrasonographic measurement of lower uterine segment to assess risk of defects of scarred uterus. Lancet 347(8997):281–284 8569360 10.1016/s0140-6736(96)90464-x

[22] Baranov A, Gunnarsson G, Salvesen K, Isberg PE, Vikhareva O (2016) Assessment of Cesarean hysterotomy scar in non‐pregnant women: reliability of transvaginal sonography with and without contrast enhancement. Ultrasound Obstet Gynecol 47(4):499–505 25720922 10.1002/uog.14833

[23] Bennich G, Rudnicki M, Wilken‐Jensen C, Lousen T, Lassen P, Wøjdemann K (2016) Impact of adding a second layer to a single unlocked closure of a Cesarean uterine incision: randomized controlled trial. Ultrasound Obstet Gynecol. 10.1002/uog.15792 26489989 10.1002/uog.15792

[24] Cahill AG, Raghuraman N, Gandhi M, Kaimal AJ (2024) First and second stage labor management: ACOG clinical practice guideline no. 8. Obstet Gynecol 143(1):144–162 38096556 10.1097/AOG.0000000000005447

[25] Di Spiezio Sardo A, Saccone G, McCurdy R, Bujold E, Bifulco G, Berghella V (2017) Risk of Cesarean scar defect following single‐vs double‐layer uterine closure: systematic review and meta‐analysis of randomized controlled trials. Ultrasound Obstet Gynecol 50(5):578–583 28070914 10.1002/uog.17401

[26] Osser OV, Valentine L (2010) Risk factors for incomplete healing of the uterine incision after caesarean section. Obstet Gynecol Surv 65(11):692 10.1111/j.1471-0528.2010.02631.x 20604776

[27] Roberge S, Demers S, Girard M, Vikhareva O, Markey S, Chaillet N et al (2016) Impact of uterine closure on residual myometrial thickness after cesarean: a randomized controlled trial. American J Obstet Gynecol 214(4):507.e1-507.e6 10.1016/j.ajog.2015.10.916 26522861

[28] Marchand GJ, Masoud A, King A, Ruther S, Brazil G, Ulibarri H et al (2021) Effect of single-and double-layer cesarean section closure on residual myometrial thickness and isthmocele—a systematic review and meta-analysis. Turkish J Obstet and Gynecol 18(4):322 10.4274/tjod.galenos.2021.71173 PMC8711674 34955322

[29] Nezhat C, Grace L, Soliemannjad R, Razavi GM, Nezhat A (2016) Cesarean scar defect: what is it and how should it be treated. Obg Manag 28(4):32–34

[30] Tarafdari A, Nazarpour M, Zargardzadeh N, Hantoushzadeh S, Parsaei M (2024) Comparing cesarean scar defect incidence after locked and unlocked repair methods among primiparous patients: a randomized double-blinded trial. J Fam Reprod Health 18(3):146 10.18502/jfrh.v18i3.16655 PMC11491697 39439735

[31] Alper E, Aksakal E, Usta I, Urman B (2024) The novel parallel closure technique compared to single-layer closure of the uterus after primary cesarean section decreases the ıncidence of ısthmocele formation and ıncreases residual myometrial thickness. Cureus. 10.7759/cureus.60932 38910631 10.7759/cureus.60932 PMC11193476

[32] Libretti A, Bracci B, De Pedrini A, Surico D, Troìa L, Remorgida V (2024) The dermabond Prineo skin closure system: benefits and complications. J Gynecol Surg 40(2):123–131

[33] Kellner H, Horky A, Louwen F, Bahlmann F, Al Naimi A (2024) The association between gynecological complaints and the uterine sonographic features in women with a history of cesarean section. Arch Gynecol Obstet 310(1):485–491 38695973 10.1007/s00404-024-07526-x PMC11169038

[34] Nithya D (2023) Effect of nutritional status and anemia in the wound healing process of post cesarean section patients. Int J Health Sci 1:5–8

[35] Özüm G, Güraslan H, Deniz L, Demirtaş T (2025) Isthmocele risk in repeated cesarean: the diagnostic and clinical role of morphometric parameters. Arch Gynecol Obstet. 10.1007/s00404-025-08238-6 41205040 10.1007/s00404-025-08238-6 PMC12705807

