# Supplementary material for: The role of cesarean section surgical techniques in the prevention of isthmocele formation: retrospective cohort study
Source: Arch Gynecol Obstet. 2026 Mar 21;313(1):139. doi: 10.1007/s00404-026-08359-6 (PMC13005875; doi:10.1007/s00404-026-08359-6)
Supplement: Supplementary file 2 — Supplementary file2 (DOCX 15 KB) [file 404_2026_8359_MOESM2_ESM.docx]

**Table 6. Comparison of Characteristics of Women Attending vs Not Attending SIS Follow-Up**

| **Variable** | | **Attended SIS follow-up (n=180)** | **Did not attend SIS follow-up (n=198)** | **p-value** |
| --- | --- | --- | --- | --- |
| **Age (years)** | | 30.8 ± 5.4 | 31.1 ± 5.6 | 0.480 |
| **BMI (kg/m²)** | | 28.6 ± 4.3 | 28.8 ± 4.4 | 0.620 |
| **Gravidity** | | 3 (1–6) | 3 (1–7) | 0.552 |
| **Parity** | | 2 (0–5) | 2 (0–5) | 0.498 |
| **Abortion** | | 0 (0–3) | 0 (0–4) | 0.960 |
| **Gestational age (weeks)** | | 38.2 ± 1.6 | 38.1 ± 1.7 | 0.731 |
| **Birth weight (grams)** | | 3220 ± 451 | 3190 ± 470 | 0.672 |
| **Preoperative anemia** | | 25.5% | 24.2% | 0.749 |
| **Cesarean section indication** | Fetal distress | 32 (33.0%) | 24 (28.9%) | 0.677 |
|  | Breech - abnormal presentation | 23 (23.7%) | 23 (27.7%) |  |
|  | Fetal growth restriction | 16 (16.5%) | 11 (13.3%) |  |
|  | Preeclampsia | 12 (12.4%) | 10 (12.0%) |  |
|  | Oligohydramnios | 13 (13.4%) | 11 (13.3%) |  |
|  | Placenta previa | 1 (1.0%) | 4 (4.8%) |  |
| **Suture technique** | Locked double-layer | 97 (53.9%) | 115 (58.1%) | 0.070 |
|  | Unlocked double-layer | 83 (46.1%) | 83 (41.9%)* |  |
